# Pyramiding Fusarium head blight resistance QTL from *T. aestivum, T. dicoccum* and *T. dicoccoides* in durum wheat

**DOI:** 10.1007/s00122-023-04426-7

**Published:** 2023-08-28

**Authors:** Rizky Pasthika Kirana, Sebastian Michel, Jose Moreno-Amores, Noemie Prat, Marc Lemmens, Maria Buerstmayr, Hermann Buerstmayr, Barbara Steiner

**Affiliations:** 1 University of Natural Resources and Life Sciences, Vienna, Department of Agrobiotechnology (IFA-Tulln), Institute of Biotechnology in Plant Production, Konrad-Lorenz-Straße 20, 3430 Tulln, Austria; 2 Laboratory of Plant Breeding, Department of Agronomy, Faculty of Agriculture, Universitas Gadjah Mada, Yogyakarta, Indonesia; 3 Plant Breeding and Genetics Section, School of Integrative Plant Science, Cornell University, Ithaca, NY USA

## Abstract

**Key message:**

FHB resistance of durum wheat was improved by introgression of *Fhb1* and resistance genes from emmer wheat and by selection against adverse alleles of elite durum wheat.

**Abstract:**

Durum wheat is particularly susceptible to Fusarium head blight (FHB) and breeding for resistance is impeded by the low genetic variation within the elite gene pool. To extend the genetic basis for FHB resistance in durum wheat, we analyzed 603 durum wheat lines from crosses of elite durum wheat with resistance donors carrying resistance alleles derived from *Triticum aestivum*, *T. dicoccum* and *T. dicoccoides*. The lines were phenotyped for FHB resistance, anthesis date, and plant height in artificially inoculated disease nurseries over 5 years. A broad variation was found for all traits, while anthesis date and plant height strongly influenced FHB severities. To correct for spurious associations, we adjusted FHB scorings for temperature fluctuations during the anthesis period and included plant height as a covariate in the analysis. This resulted in the detection of seven quantitative trait loci (QTL) affecting FHB severities. The hexaploid wheat-derived *Fhb1* QTL was most significant on reducing FHB severities, highlighting its successful introgression into several durum wheat backgrounds. For two QTL on chromosomes 1B and 2B, the resistance alleles originated from the *T. dicoccum* line Td161 and *T. dicoccoides* accessions Mt. Hermon#22 and Mt. Gerizim#36, respectively. The other four QTL featured unfavorable alleles derived from elite durum wheat that increased FHB severities, with a particularly negative effect on chromosome 6A that simultaneously affected plant height and anthesis date. Therefore, in addition to pyramiding resistance genes, selecting against adverse alleles present in elite durum wheat could be a promising avenue in breeding FHB-resistant durum wheat.

**Supplementary Information:**

The online version contains supplementary material available at 10.1007/s00122-023-04426-7.

## Introduction

Fusarium head blight (FHB) is one of the most destructive diseases of bread wheat (*Triticum aestivum* L.) and durum wheat (*Triticum durum* Desf.) and is caused by fungi of the genus *Fusarium* (Khan et al. 2020). The accumulation of mycotoxins in grains due to this disease (Góral et al. 2018) is imperiling food and feed safety and imposes risks to human and animal health (Da Rocha et al. 2014). Breeding of FHB-resistant wheat cultivars is regarded the most sustainable way to control this disease. Resistance to FHB is a quantitatively inherited trait influenced by environmental factors with significant genotype-by-environment interactions. In bread wheat, more than 400 FHB resistance QTL have been reported, mapping across all 21 chromosomes (Ma et al. 2020; Buerstmayr et al. 2020), including major QTL like *Fhb1* (Waldron et al. 1999; Anderson et al. 2001), *Fhb*2 (Cuthbert et al. 2007), *Fhb3* (Qi et al. 2008), *Fhb4* (Xue et al. 2010), *Fhb5* (Xue et al. 2011), *Fhb6* (Cainong et al. 2015), and *Fhb*7 (Guo et al. 2015). On the other hand, only a few FHB resistance QTL with only small effects have been identified so far in durum wheat QTL mapping studies (Prat et al. 2014; Haile et al. 2019; Buerstmayr et al. 2020).

In general, resistance breeding in durum wheat is impeded by the low genetic variation in the elite gene pool, with most durum wheat cultivars being moderately to highly susceptible (Clarke et al. 2010; Miedaner and Longin 2014). Introgression of resistance gene(s) into durum wheat is a promising way to enlarge the durum resistance gene pool, and the search for genetic material that could improve this trait has led to wild and cultivated relatives of durum wheat. Prat et al. (2017) reported the successful introgression of *Fhb1*, the most stable resistance QTL located on chromosome 3B of bread wheat, into *T. durum*. Other studies described resistance alleles derived from cultivated emmer wheat *T. dicoccum* (Buerstmayr et al. 2012; Ruan et al. 2012; Zhang et al. 2014), wild emmer wheat *T. dicoccoides* (Otto et al. 2002; Gladysz et al. 2007; Chen et al. 2007; Buerstmayr et al. 2013), Persian wheat *T. carthlicum* (Somers et al. 2006; Sari et al. 2018), and *Thinopyrum elongatum* (Kuzmanović et al. 2019) that have the potential to increase the FHB resistance in *T. durum*.

Detected resistance QTL often overlapped with genes controlling phenological or morphological traits, such as heading/flowering date and plant height (Buerstmayr et al. 2012; Giancaspro et al. 2016; Miedaner et al. 2017; Prat et al. 2017; Steiner et al. 2019). These traits may impact fungal infection and disease spread and should be evaluated for meaningful interpretation of FHB resistance QTL results to detangle genetically controlled, active or passive resistance mechanisms, from environmental influences.

Stacking QTL enhances resistance to FHB, as shown in many studies with hexaploid wheat (Miedaner et al. 2006; Buerstmayr et al. 2020; Zhang et al. 2021; Ghimire et al. 2022) and pyramiding the major QTL *Fhb1*, *Fhb4*, and *Fhb*5 for example reduced the disease severity by up to 95% (Zhang et al. 2021). Hence, introducing resistance genes from various genetic resources is also expected to increase the FHB resistance of durum wheat. Using statistical genetics methods such as genome-wide association mapping (GWAS) has shown large promise in detecting resistance QTL in various genetic resources of durum wheat (Miedaner et al. 2017; Steiner et al. 2019; Ruan et al. 2020). The ability to capture more recombination events makes GWAS a powerful exploratory analytical tool for understanding genetic variation in panels with diverse genetic backgrounds, and the detected QTL can subsequently be used in downstream applications like marker-assisted selection (Alqudah et al. 2020).

In this study, we analyzed ten related multi-parental durum wheat populations derived from crosses between elite durum wheat and resistance donors carrying resistance allele introgressions from the *T. aestivum*, *T. dicoccum*, and *T. dicoccoides* gene pools. The objectives were thus to develop breeding material with improved resistance and elucidate the genetic control and relationships of FHB resistance with anthesis date and plant height in this germplasm.

## Materials and methods

### Plant materials and experimental design

A total of 603 durum wheat recombinant inbred lines (Table S1a) developed at the University of Natural Resources and Life Sciences, Vienna, Department of Agrobiotechnology (IFA-Tulln) was used in this study. According to the parents the lines were grouped into ten subpopulations (Table 1). All lines are F_7_ derivatives of four-way crosses whereof 153 lines (subpopulations 1–3) include two elite durum lines and two resistant donor lines, 361 lines (subpopulations 5–10) originate from crosses of one elite durum wheat cultivar and three resistance donors and subpopulation 4, comprising 89 lines, descents from crosses of four resistance donors.

**Table 1:** Description of the plant material, including subpopulation, number of lines, parents of the four-way crosses, and resistance alleles donors

Subpopulation	No. of lines	Parental lines					
		Elite durum wheat		Resistance donors carrying FHB resistance alleles from*:*			
				*T. aestivum* (Sumai-3, *Fhb1*)	*T. dicoccum* (Td161)	*T. dicoccoides* (Mt. Hermon#22; Mt. Gerizim#36)	
1	53	Karur	SZD1029K	DBC480	Heli94		
2	50	Karur	SZD1029K	DBC480		I19.94	
3	50	Durobonus	SZD1029K		Heli94	I19.94	
4	89			DBC480	Heli123	I19.32	I17.122
5	41	Floradur		DBC480		I19.32	I18.14
6	23	Durobonus		DBC480		I19.32	I18.14
7	50	Karur		DBC480		I19.32	I18.14
8	49	Durobonus		DBC480	Heli123	I19.32	
9	103	Floradur		DBC480	Heli123	I19.32	
10	95	Karur		DBC480	Heli31	I19.39	

The durum wheat resistance donors carried resistance alleles from *T. aestivum* (DBC480), *T. dicoccum* (Heli31, Heli94, and Heli123), and *T. dicoccoides* (I17, I18, and I19) and were developed at the University of Natural Resources and Life Sciences, Vienna (Tables 1, S1b). DBC480 is a BC_4_ line between the highly resistant *T. aestivum* cultivar Sumai-3 and the Austrian *T. durum* cultivar Semperdur as the recurrent parent, and carries the major FHB resistance QTL *Fhb1* (Prat et al. 2017). Heli31, Heli94, and Heli123 are BC_1_ sister lines from crosses between the resistant *T. dicoccum* line Td161 and the susceptible durum wheat cultivar Helidur as recurrent parent (Buerstmayr et al. 2012). I17, I18, and I19 are BC_1_ lines with Helidur as recurrent parent, whereby I17 and I18 have the *T. dicoccoides* lines Mt. Hermon#22 (aka *T. dicoccoides* 1A) and I19 has Mt. Gerizim#36 (aka *T. dicoccoides* 52A) as FHB resistance source (Gladysz et al. 2007; Buerstmayr et al. 2013).

Elite parental lines were the French cultivar Karur (registered 2002 by RAGT), the Austrian durum wheat cultivars Durobonus (registered 2004 by Saatzucht Donau GesmbH & Co KG) and Floradur (registered 2003 by Saatzucht Donau GesmbH & Co KG) as well as the Austrian breeding line SZD1029K (Saatzucht Donau GesmbH & Co KG). All elite durum wheat parents possess the semi-dwarfing allele *Rht-B1b*, while all resistance donors harbor the *rht-B1a* wildtype allele (Buerstmayr et al. 2012, 2013; Prat et al. 2017).

The lines were tested in nine artificially inoculated field trials at the University of Natural Resources and Life Sciences, Vienna, Department of Agrobiotechnology (IFA-Tulln), Austria (latitude 48°20′0″ N, longitude 16°3′0″ E, altitude 177 m), following crop management standards described by Buerstmayr et al. (2002). Each line was thereby phenotyped in five of the nine trials conducted between 2012 and 2020 (Table 2). All trials were sown in early March and arranged as randomized complete block designs, with one to three blocks and single or double-row plots (Table 2). Rows were 1 m in length with a row spacing of 17 cm for double rows. Blocks were sown 1–2 weeks apart depending on the weather conditions, which led to a mild anthesis delay of a few days in late-sown blocks.

**Table 2:** Tested plant material, plot, and block information of each experimental year

Year	Tested subpopulations	Plot^a^	Number of blocks
2012	1, 2, 3	SR	1
2013	1, 2, 3	SR	2
2014	1, 2, 3	DR	3
2015	1, 2, 3	DR	3
2016	4, 5, 6, 7, 8, 9, 10	SR	1
2017	4, 5, 6, 7, 8, 9, 10	SR	2
2018	4, 5, 6, 7, 8, 9, 10	SR	2
2019	1, 2, 3, 4, 5, 6, 7, 8, 9, 10	DR	2
2020	4, 5, 6, 7, 8, 9, 10	DR	2

^a^*SR* Single row, *DR* Double row

### Fusarium inoculation and phenotyping

A macroconidia suspension of a single spore isolate of *F. culmorum* 'Fc91015' was prepared and stored at − 80 °C (Buerstmayr et al. 2000) until it was used for artificial spray inoculation of the field trial. The spray inoculation started when the earliest plot of a block reached mid-anthesis and repeated every second day until the last plot reached mid-anthesis. The inoculum aliquots were thawed in lukewarm water shortly before inoculation and diluted to a conidia concentration of 12.5 × 10^3^ ml^−1^. Inoculations were carried out by spraying about 100 ml m^−2^ of diluted conidia suspension onto the heads using a battery-driven backpack sprayer in the late afternoon. An automatic mist irrigation system based on leaf moisture measurement was employed to control humidity in the field trials for the first 20 h after each inoculation in order to promote infection with *F. culmorum*.

The anthesis date was recorded and converted into the number of days after May 1st, and plant height was measured in centimeters for each plot. FHB severity was visually estimated as the percentage of infected spikelets within each plot on days 10, 14, 18, 22, 26, and 30 after anthesis. An integrated measure of FHB severity was determined by calculating the area under the disease progress curve (AUDPC_uncorrected_) based on the formula delineated by Buerstmayr et al. (2000).

Correction of each FHB scoring by anthesis date and accumulated thermal time was done as described in Moreno-Amores et al. (2020) to avoid ambiguity of the FHB severity due to the influence of environmental and plant phenological factors. Briefly, a feature selection model, i.e., lasso regression (Moreno-Amores et al. 2020) was fitted using anthesis date and relevant accumulated thermal time variables as predictors of FHB severity, and the new set of predictors served to recalculate AUDPC (AUDPC_corrected_).

### Statistical analysis of the phenotypic data

FHB severities (AUDPC_uncorrected_, AUDPC_corrected_), date of anthesis (defined in days after May 1st) and plant height (cm) were used for the analysis. Single trials were analyzed for each trait with the linear mixed model:

$${\mathcal{Y}}_{ik}= \mu + {g}_{i} +{r}_{k}+{\varepsilon }_{ik}$$

where ${\mathcal{Y}}_{ik}$ denotes the observation of the individual plot, $\mu$ is the grand mean, ${g}_{i}$ is the genetic effect of the $i$th genotype, ${r}_{k}$ is defined as the effect of the *k*th replication (block), and ${\varepsilon }_{ik}$ is the residual term. Variance components of all experiments combined were determined by applying the following linear mixed model:

$${\mathcal{Y}}_{ijk}= \mu + {g}_{i}+{e}_{j}+{e}_{j}({r}_{k})+{(\mathrm{ge})}_{ij}+{\varepsilon }_{ijk}$$

where ${\mathcal{Y}}_{ijk}$ is the phenotypic record of the $i$th genotype tested at the $j$th year in the $k$th replication, $\mu$ is the grand mean and ${g}_{i}$ is the genetic effect of the $i$th genotype. The environment effect ${e}_{j}$ is defined as the effect of the $j$th year, ${e}_{j}{(r}_{k})$ is the effect of the $k$th replication within the $j$th year, $({\mathrm{ge})}_{ij}$ describes the genotype-by-environment interaction, and ${\varepsilon }_{ijk}$ is the residual term.

All effects were considered to be random, except ${g}_{i}$ which was modeled as a fixed effect to obtain the best linear unbiased estimates (BLUEs).

Heritabilities were estimated by modeling all effects as random, and using the variance components determined by the restricted maximum likelihood method following the formula (Holland et al. 2003):

$${\rm H}^{2}={\sigma }_{G}^{2}/({\sigma }_{G}^{2} +{\sigma }_{\mathrm{GY}}^{2}/y+{\sigma }_{\upvarepsilon }^{2}/yr)$$

where ${\sigma }_{G}^{2}$ is the genetic variance, ${\sigma }_{\mathrm{GY}}^{2}$ the genotype by year interaction, ${\sigma }_{\upvarepsilon }^{2}$ the residual variance component, $y$ the number of years, and $r$ the number of replications. Pearson correlation coefficients of FHB severity (AUDPC_uncorrected_, AUDPC_corrected_), anthesis date, and plant height were estimated for all pairwise trial combinations, and correlations among the traits were calculated for BLUEs across all trials and for each trial individually. All statistical analyses of the phenotypic data were performed with R 3.5.1 (R Core Team 2020) using the package *sommer* (Covarrubias-Pazaran 2016).

### Genotyping and marker data

DNA of each line of the population and parental and grandparental lines was extracted from fresh leaves using a modified CTAB-based procedure (Saghai-Maroof et al. 1984). High-density genotyping of all individuals was performed using genotyping-by-sequencing with the DArTseq platform (DArT PL, Canberra, Australia). Markers identified by the DArTseq assay comprise SNPs as well as presence-absence variations (PAV) (Sansaloni et al. 2011; Li et al. 2015). Using the R package *dartR 2.0.3* (Gruber et al. 2018), markers were filtered based on a reproducibility ≥ 95%, and all monomorphic loci were filtered out. Furthermore, markers with ≥ 5% missing data and ≥ 5% heterozygotes were removed. Additionally, all lines were genotyped with three specific markers: UMN10 (Liu et al. 2008) and the Kompetitive Allele Specific PCR (KASP) marker 46 (Schweiger et al. 2016), which are known to be linked with the major resistance QTL *Fhb1*, and a marker diagnostic for the semi-dwarfing *Rht-B1* gene (Ellis et al. 2002). The DArTseq SNPs, KASP46, and *Rht-B1* markers were coded as 1, 0, − 1, which correspond with three genotypes of a single SNP: 1 = homozygous (AA), 0 = heterozygous (AB), − 1  = homozygous (BB). The DArTseq PAV and UMN10 were coded as 1 (presence) and − 1 (absence). The physical positions of the markers were determined by aligning the marker sequences to the *T. turgidum* durum wheat Svevo (RefSeq Rel. 1.0) reference genome (Priyam et al. 2019) with a basic local alignment search tool (BLAST). Marker imputation for all missing data was done using the R package *missForest* (Stekhoven and Bühlmann 2012) by considering the chromosome information for each crossing. After removing markers with a minor allele frequency < 5%, a total of 18,763 high-quality polymorphic markers were available for further analysis with 2.5% of the data being imputed.

### Population structure and linkage disequilibrium analysis

We conducted a principal component analysis (PCA) of the filtered and imputed genotypic data with the R function *prcomp* to appraise the population structure. Including the subpopulations information, the PCA was plotted using the R package *ggplot2* (Wickham 2016). The pairwise marker linkage disequilibrium (LD) of each chromosome was measured as the squared allele frequency correlations (*r*^2^) as described in Weir (1996) using the R package *LDheatmap* (Shin et al. 2006). The LD decay based on the marker matrix and the map with distances between markers in mega base pairs (Mbp) was generated using the package *sommer* (Covarrubias-Pazaran 2016).

### Genome-wide association mapping

Genome-wide association mapping was performed with the R package *sommer* (Covarrubias-Pazaran 2016) by fitting the following mixed linear model for each individual trait:

$$\mathcal{Y}=\mathcal{X}b+Mi +\mathcal{Z}g+e$$

where $\mathcal{Y}$ is a vector of across trials BLUEs of observed traits and $\mathcal{X}$ is the fixed effect design matrix, where $b$ is a vector of fixed effects that included the grand mean, while plant height was modeled as an additional fixed covariate in the analysis of FHB severity. $M$ is the vector of alleles for a given marker, and $i$ is a vector of genetic effects explained by this individual marker. $g$ is a vector of additive genetic effects of the genotypes following $g \sim \mathrm{ N}(0, G{\sigma }_{A}^{2})$, with the genetic relationship matrix $G$ and the corresponding design matrix $\mathcal{Z}$. A QTL was considered significant at the threshold of adjusted false discovery rate (FDR) of *α *= 0.05. Quantile–quantile (Q–Q) plots based on observed and expected − log_10_ *p* values were drawn using the R package *qqman* (Turner 2018) to check the efficiency of the model to control for genetic relatedness, population stratification and spurious marker-trait associations (Yu et al. 2006). Furthermore, a stepwise regression analysis was conducted with the markers that passed the significance threshold to determine a non-redundant set of the most significant marker-trait associations. Genetic variations explained by each marker were calculated using the sum of squares of analysis of variance of all significant markers involving plant height as a covariate for FHB severity (AUDPC_corrected_) data. Additive effects were estimated as half the difference between the averages of the BLUEs value of the homozygote for each marker.

## Results

### Phenotypic variations and trait correlations

The experimental durum wheat population displayed large and continuous variations for FHB severity (AUDPC_uncorrected_), date of anthesis and plant height but with high correlations between FHB severity and anthesis date (*r *= 0.47**) and plant height (*r *=  − 0.64**), such that later flowering and shorter lines tended to have higher disease severities (Table 3, Fig. S1). Corrected FHB severities (AUDPC_corrected_) considering anthesis date and thermal accumulation during the evaluation period nearly eliminated the association of FHB severity with anthesis date (*r *=  − 0.06**) and also reduced the significant negative correlation between FHB severity and plant height (*r *=  − 0.45**) (Tables 3, S2).

**Table 3:** Pearson correlations between best linear unbiased estimates (BLUEs) across years of FHB severity (AUDPC_uncorrected_ and AUDPC_corrected_) with anthesis date and plant height across populations and within subpopulations

Population	FHB severity (AUDPC_uncorrected_)		FHB severity (AUDPC_corrected_)^a^	
	Anthesis date	Plant height	Anthesis date	Plant height
Across populations	0.47^**^	− 0.64^**^	− 0.06^ns^	− 0.45^**^
*Subpopulations*				
1	0.35^**^	− 0.86^**^	0.06^ns^	− 0.90^**^
2	0.49^**^	− 0.73^**^	0.27^ns^	− 0.73^**^
3	0.38^**^	− 0.73^**^	0.08^ns^	− 0.73^**^
4	0.50^**^	− 0.66^**^	− 0.13^ns^	− 0.52^**^
5	0.45^**^	− 0.52^**^	0.08^ns^	− 0.56^**^
6	0.58^**^	− 0.79^**^	0.27^ns^	− 0.76^**^
7	0.65^**^	− 0.59^**^	0.21^ns^	− 0.72^**^
8	0.36^*^	− 0.54^**^	− 0.38**	− 0.63^**^
9	0.62^**^	− 0.65^**^	0.16^ns^	− 0.58^**^
10	0.55^**^	− 0.55^**^	0.04^ns^	− 0.53^**^

Significance codes: **p < 0.01, *p < 0.05, ^ns^not significant

^a^AUDPC corrected for anthesis date and accumulated thermal time according to Moreno-Amores et al. (2020)

**Fig. 1:**
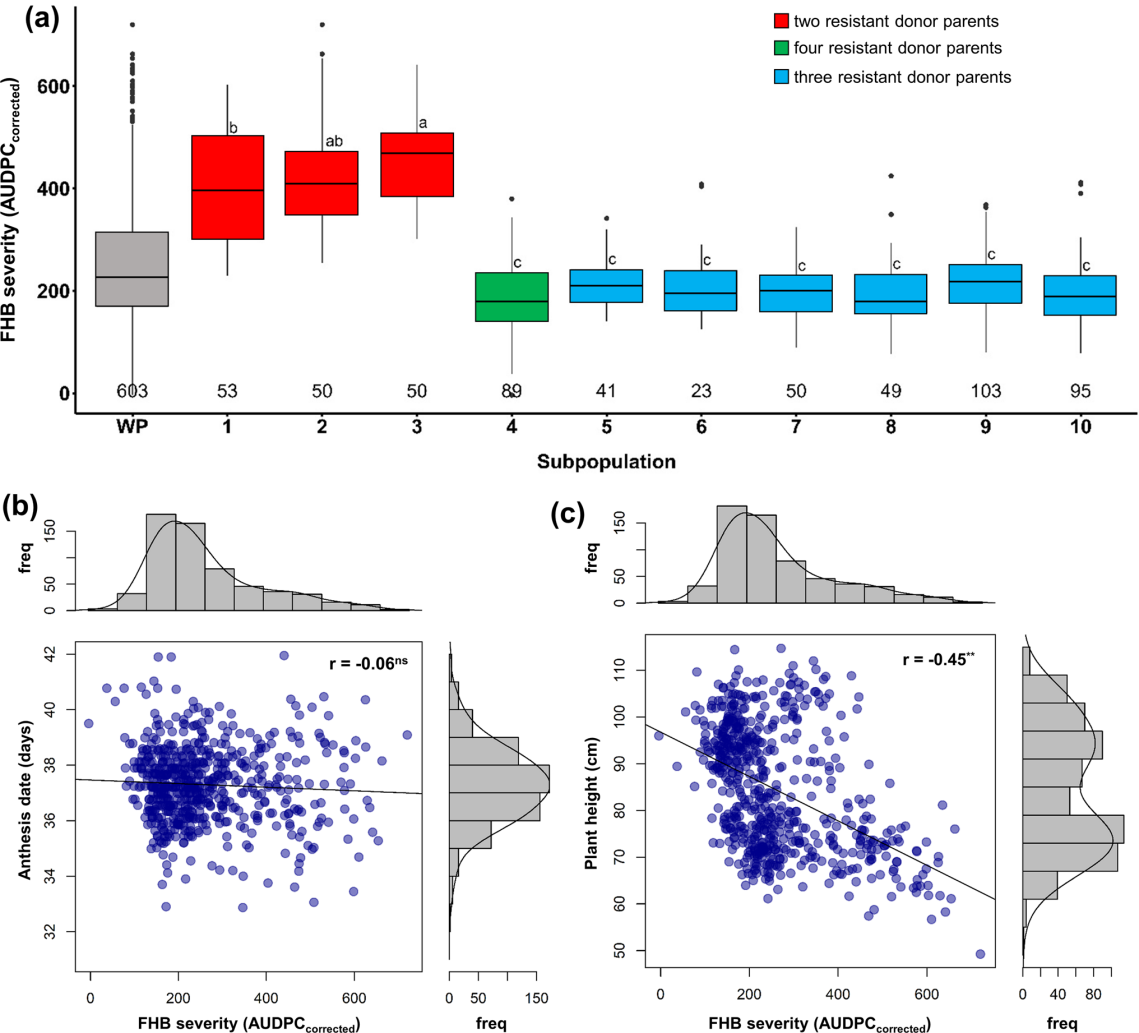
**a** Boxplots of best linear unbiased estimates (BLUEs) across years for FHB severity (AUDPC_corrected_) after thermal accumulation correction across populations (whole population (WP)) and for individual subpopulations, the groups with different index letters are significantly different at *p *< 0.05 based on Tukey test, the numbers above the x-axis line indicate the number of lines; Scatter plots and marginal histograms of frequency distribution for FHB severity (AUDPC_corrected_) against **b** anthesis date (number of days from May 1st) and **c** plant height (cm)

Given the counterbalancing effect of the ‘thermal accumulation correction’ on the influence of anthesis date on disease severities, AUDPC_corrected_ was used as the measure for FHB severity for all further analysis. The 603 durum wheat lines displayed large and continuous variation for FHB severity, which was slightly skewed toward resistance with an AUDPC mean of about 270 (Fig. 1, Table 4). We identified many moderately resistant lines, only subpopulations 1, 2, and 3, that had two elite lines in their pedigree were significantly more diseased compared to subpopulations 4 to 10, that had either one or no elite line in their pedigrees (Fig. 1a, b). The higher susceptibility of the three subpopulations was also evident in 2019 when all ten subpopulations were evaluated in the same environment.

**Table 4:** Means, minimum and maximum values of best linear unbiased estimates (BLUEs) for FHB severity (AUDPC_corrected_), anthesis date (number of days from May 1st) and plant height (cm) of the population and parents and variance components, broad-sense heritability coefficient (*H*^2^), and least significant differences (LSD *α* < 0.05)

Population	FHB severity (AUDPC_corrected_)			Anthesis date (number of days after May 1st)			Plant height (cm)		
	Min	Mean	Max	Min	Mean	Max	Min	Mean	Max
	1.20	266.58	725.36	27.00	37.03	52.00	40.00	83.98	142.00
*Variance* ± *SE*									
$\sigma_{G}^{2}$		51,077.20 ± 25,728.90			1.64 ± 0.12			165.34 ± 10.03	
$\sigma_{{{\text{GY}}}}^{2}$		3920.00 ± 250.40			0.73 ± 0.08			19.67 ± 1.34	
$\sigma_{{\upvarepsilon }}^{2}$		6681.50 ± 188.70			3.24 ± 0.09			40.37 ± 1.08	
*H*^*2*^		0.84			0.78			0.95	
LSD(5%)		158.71			1.97			7.11	
*Parents*									
Resistance donors									
DBC480	0.80	127.01	402.69	32.00	37.06	48.00	70.00	116.24	145.00
Heli123	263.75	263.75	263.75	46.00	46.50	47.00	42.00	60.00	85.00
Heli31	192.65	237.90	337.52	35.00	36.00	38.00	85.00	92.50	102.00
Heli94	11.96	118.06	448.84	34.00	39.75	50.00	75.00	114.59	140.00
I17.122	184.35	257.50	360.98	35.00	35.50	36.00	80.00	82.75	90.00
I18.14	151.16	210.03	276.40	33.00	34.50	36.00	70.00	95.75	115.00
I19.32	34.45	363.83	1095.04	27.00	37.02	46.00	45.00	81.48	142.00
I19.94	61.95	227.02	763.34	32.00	38.62	50.00	65.00	99.31	150.00
Elite durum wheat									
Durobonus	129.24	448.44	1020.94	32.00	36.35	48.00	50.00	68.85	85.00
Floradur	184.35	263.27	382.99	35.00	36.00	38.00	80.00	85.00	89.00
Karur	205.80	446.08	780.00	32.00	37.06	48.00	60.00	75.71	95.00
SZD1029K	176.06	601.36	940.00	34.00	39.69	48.00	50.00	59.90	70.00

The genotypic variance was much larger than the genotype-by-year interaction, yielding a high across-year broad-sense heritability for FHB severity (*H*^*2*^ = 0.84, Tables 4 and S3). The analysis of variance revealed that all sources of variation had highly significant effects on FHB severity, with the genotype being the most important factor (Table S4). Pearson correlation coefficients for FHB severity between trials were positive, ranging from *r *= 0.18–0.76 (Table S5).

Anthesis date and plant height also revealed large variations. The anthesis period lasted 25 days, although the range within subpopulations was smaller (Tables 4, S3). Similarly, large variation in plant height was apparent, ranging between 40 and 142 cm (Tables 4, S3), and showed a bimodal frequency distribution (Fig. 1). High heritabilities of *H*^2^ = 0.78 for anthesis date and *H*^2^ = 0.95 for plant height confirmed the data quality as being adequate for further analysis (Table 4). BLUEs for all investigated traits for the individual trials and across all trials are summarized in Tables S6 and S7.

### Population structure and linkage disequilibrium

**Fig. 2:**
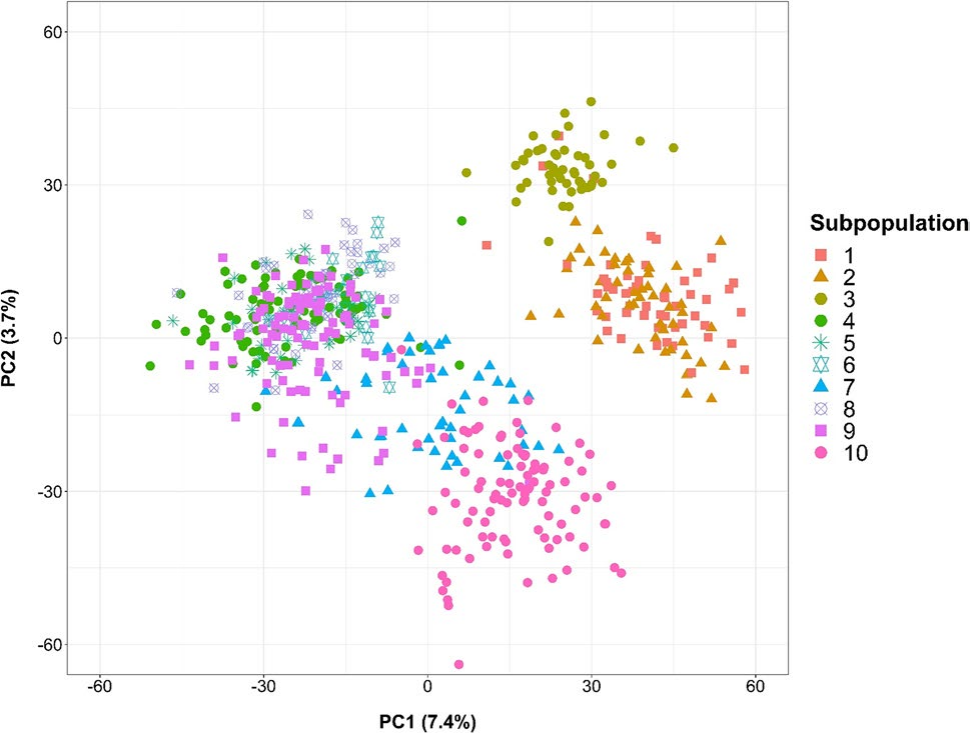
Principal component analysis based on genotype data of 603 durum wheat lines with the % of variation explained by the first principal component (PC1) and the second principal component (PC2). Individual values described by the same symbol and color are belonging to the same subpopulation

In this study, 13,640 markers were distributed over the 14 durum wheat chromosomes and used for population structure analysis and GWAS (Table S8). The PCA analysis divided the 603 durum wheat lines into two main distinct clusters based on the number of elite durum wheat cultivars used for crossing (Fig. 2). The two clusters were further delineated into subclusters based on subpopulations, but these could not be genetically separated. The first two axes explained only 11.1% of the variation (7.4 and 3.7% for axis 1 and 2, respectively) confirming the lack of a pronounced population structure in the experimental durum wheat population.

The LD patterns of the 603 durum wheat lines are graphically displayed by scatter plots of pairwise LD (*r*^2^) over physical distance in Mbp (Figs. S2, S3). The intrachromosomal LD decay, below a *r*^2^ critical threshold lower than 0.2, showed a mean value of 39 Mbp for the whole genome (Fig. S2) and varied between chromosomes from 16.02 Mbp (chromosome 6A) to 103.5 Mbp (chromosome 2A) (Fig. S3).

### Association mapping and QTL identification for FHB resistance

**Fig. 3:**
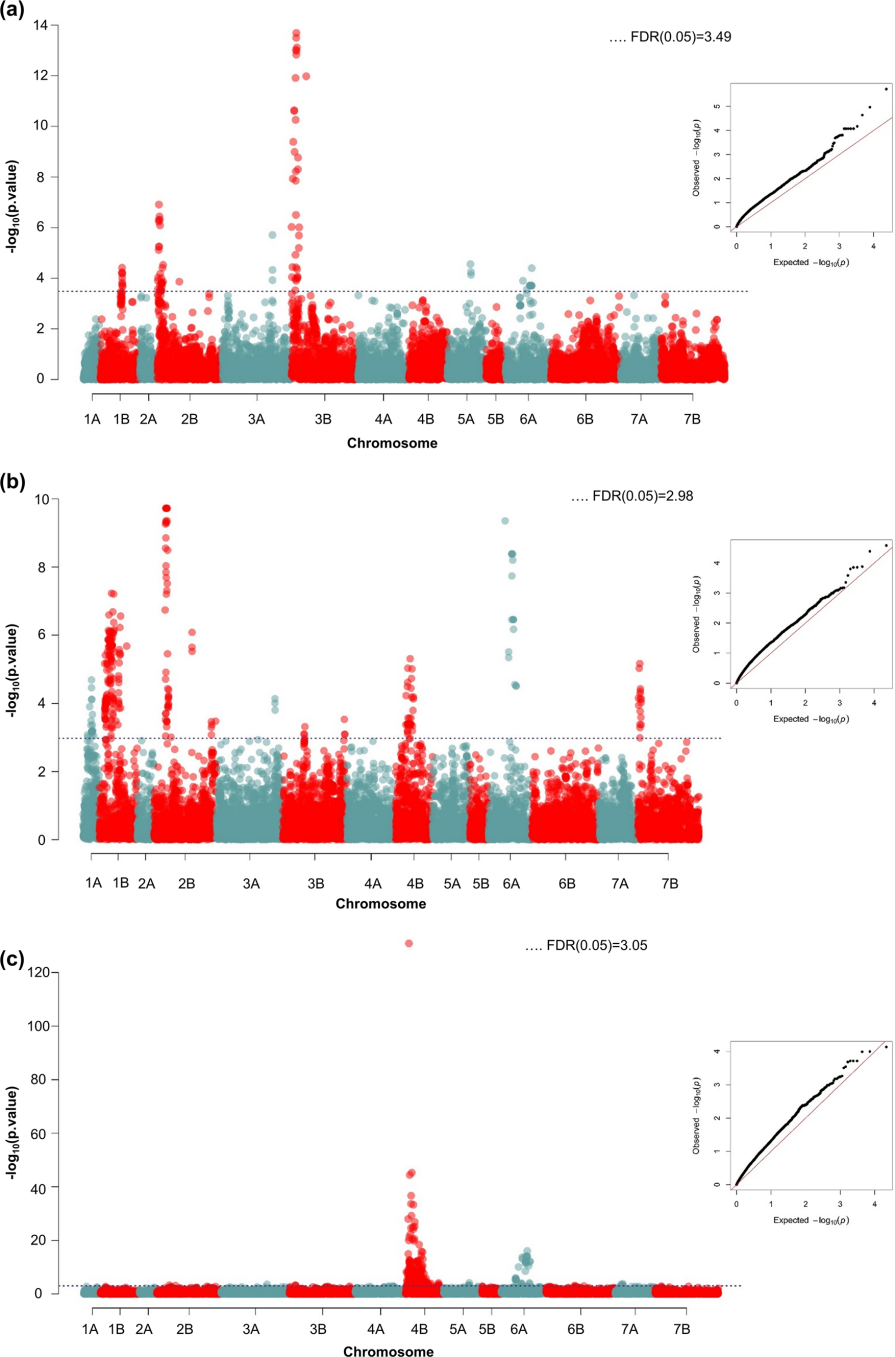
Manhattan and Q–Q plots of the SNP associations with best linear unbiased estimates (BLUEs) for **a** FHB severity (AUDPC_corrected_) after thermal accumulation correction and plant height as a covariate, **b** anthesis date and **c** plant height in the durum wheat population across the 2012–2020 trials. The horizontal dotted line shows the − log10 *p* value with FDR 5% significant threshold

Seven QTL had a significant association with FHB severity when correcting for anthesis date by thermal accumulation and including plant height as a covariate in the GWAS model (Table 5, Fig. 3a). The resistance donors contributed beneficial FHB resistance alleles at three QTL located on chromosomes 1B, 2B and 3B. The other four significant associations detected QTL on chromosomes 3A, 3B, 5A and 6A, at which elite durum wheat cultivars carried unfavorable alleles that increased FHB severity. The most significant association was revealed for the *Fhb1*-specific KASP marker 46 located on chromosome 3B; it explained 10% of the genetic variance and had a pronounced additive effect on reducing FHB severities but no significant effects on anthesis date and plant height. In addition, resistance QTL were discovered on chromosome 1BL (marker 1,719,911) for which the favorable allele originated from parental lines with a *T. dicoccum* genetic background and on chromosome 2BS (marker 1,723,994) with *T. dicoccoides* parental lines as the donors. Haplotype comparisons for markers in the QTL intervals revealed for the ‘1B QTL’ *T. dicoccum* line Td161 and for the ‘2B QTL’ the *T. dicoccoides* lines Mt. Hermon#22 (aka *T. dicoccoides* 1A) and Mt. Gerizim#36 (aka *T. dicoccoides* 52A) as the sources of resistance.

**Table 5:** Markers associated with best linear unbiased estimates (BLUEs) for FHB severity (AUDPC_corrected_) using plant height as a covariate, anthesis date (number of days from May 1st) and plant height (cm); their chromosomal and physical positions, − log10 *p* values, allele frequencies and allele donors, explained genetic variance and additive effects

Marker	Chromosome	Position (base pair)	− log_10_ *p* value	Frequency of allele	Allele donor^a^	Explained genetic variance (%)	Additive effect^b^		
							FHB severity	Anthesis date	Plant height
*FHB severity*									
1,719,911	1B	505,559,153	4.20	0.12	Heli123, Heli94, (Td161)	7.66	− 22.38		
1,723,994	2B	13,308,290	3.79	0.24	I17.122, I18.14, I19.32, I19.8 (Mt. Hermon#22; Mt. Gerizim#36)	21.20	− 43.80		
6,042,974	3A	638,032,203	3.93	0.14	Floradur, SZD1029K	13.01	45.51		
KASP46	3B	10,890,613	13.11	0.25	DBC480 (Sumai3, *Fhb1*)	10.28	− 26.97		
4,003,681	3B	11,935,133	4.03	0.28	Durobonus, I19.94 (Mt. Gerizim#36), Karur, SZD1029K	5.81	62.44		
1,021,609	5A	540,667,162	4.24	0.47	DBC480 (Semperdur), Durobonus, Karur, SZD1029K	7.37	28.25		
1,087,772	6A	326,642,408	4.40	0.07	SZD1029K	12.88	128.29	0.31	− 8.27
*Anthesis date*									
4,009,175	1A	389,684,753	4.37	0.28	Durobonus, I18.14, I19.32, I19.8, I19.94	5.25		− 0.34	
1,009,767	1B	519,883,466	6.10	0.12	Heli123	3.44		0.50	
1,200,783	2B	54,217,790	7.05	0.09	Durobonus, Karur	17.88		− 0.68	
4,394,159	2B	622,240,148	5.90	0.21	Heli94, Karur, SZD1029K	1.95		0.16	
1,075,069	3A	712,118,115	3.99	0.24	Heli31, Heli94, I17.122, I18.14, I19.39	4.26		− 0.24	
1,382,159	3B	827,589,239	3.07	0.08	Heli123	1.24		0.37	
1,720,461	3B	180,878,626	3.31	0.12	Heli123, Heli31	6.13		0.39	
1,308,306	4B	391,662,089	5.13	0.29	DBC480, Durobonus, Heli31, Heli94, I17.122, I18.14, I19.8, Karur	3.71		− 0.32	
49,090,444	4B	220,694,302	4.55	0.06	Heli123	1.48		0.55	
1,087,772	6A	326,642,408	6.34	0.07	SZD1029K	5.85	128.29	0.31	− 8.27
1,126,624	7B	2,340,299	4.87	0.07	Heli123, I19.39	2.35		0.55	
*Plant height*									
Rht-B1	4B	29,294,104	127.61	0.48	Durobonus, Floradur, Heli123, Heli31, I17.122, I19.32, Karur, SZD1029K	86.91	30.43		− 10.38
1,087,772	6A	326,642,408	16.03	0.07	SZD1029K	33.17	128.29	0.31	− 8.27

^a^For the resistance donors, the sources of FHB resistance derived from grandparents are given in brackets

^b^Positive additive effects increase FHB severities, delay anthesis and increase plant height; negative additive effects reduce FHB severities, cause earlier anthesis and reduce plant height

The four QTL on chromosomes 3A, 3B, 5A, and 6A detected susceptibility alleles in elite durum wheat that increased FHB severities. Several elite durum wheat parents inherited the unfavorable alleles for the significant marker associations on chromosomes 3A, 3B, and 5A (Table 5). Notable, the very short and highly susceptible elite durum wheat SZD1029K contributed at all loci the unfavorable allele. The susceptibility allele on chromosome 6A (marker 1,087,772) showed low allele frequency and was unique for SZD109K. Consequently, it was only identified in subpopulations 1, 2, and 3 that had higher FHB severities than the other subpopulations (Fig. 1a). The 6A susceptibility allele increased FHB severities significantly and at the same time delayed anthesis (0.31 day) and reduced plant height (− 8.27 cm) (Table 5). All other six markers associated with FHB severity had no significant effects on anthesis date.

Adjusting FHB severities for anthesis date and accumulated temperature and using plant height as a covariable diminished colocalization of markers associated with FHB severity and simultaneously with anthesis date or plant height. Particularly evident is the corrective effect on the semi-dwarfing locus *Rht-B1*, which was the predominant regulator of FHB severities but completely disappeared when using plant height as a covariable (Fig. S4, Table S9). Nevertheless, the resistance QTL derived from hexaploid wheat, *Fhb1*, was significantly associated with FHB severity regardless of statistical adjustment for plant height or temperature correction on AUDPC values, confirming its robustness and importance.

### Marker-trait associations for anthesis date and plant height

Anthesis date was controlled by eleven QTL located on chromosomes 1A, 1B, 2B, 3A, 3B, 4B, 6A, and 7B. All detected associations had minor to medium additive effects, the QTL on chromosome 2B (marker 1,200,783) was the most significant, revealed the highest explained genetic variance (17.88%) and colocalized with the major determinant of anthesis date *Ppd-B1* (Würschum et al. 2019) (Fig. 3b, Table 4).

Plant height was regulated by two loci with major effects. The diagnostic marker for the semi-dwarfing locus *Rht-B1* detected the most prominent association (− log_10_ *p* value 127.61) and a large additive effect, the 'short' *Rht-B1b* allele reduced plant height substantially by − 10.38 cm (Table 4, Fig. 3c). Likewise, the second locus on chromosome 6A (marker 1,087,772) significantly reduced plant height (− 8.27 cm) but also had a significant effect on anthesis date and both loci colocalized with QTL for FHB severities (Table 4).

### Combined QTL effects on FHB severities

**Fig. 4:**
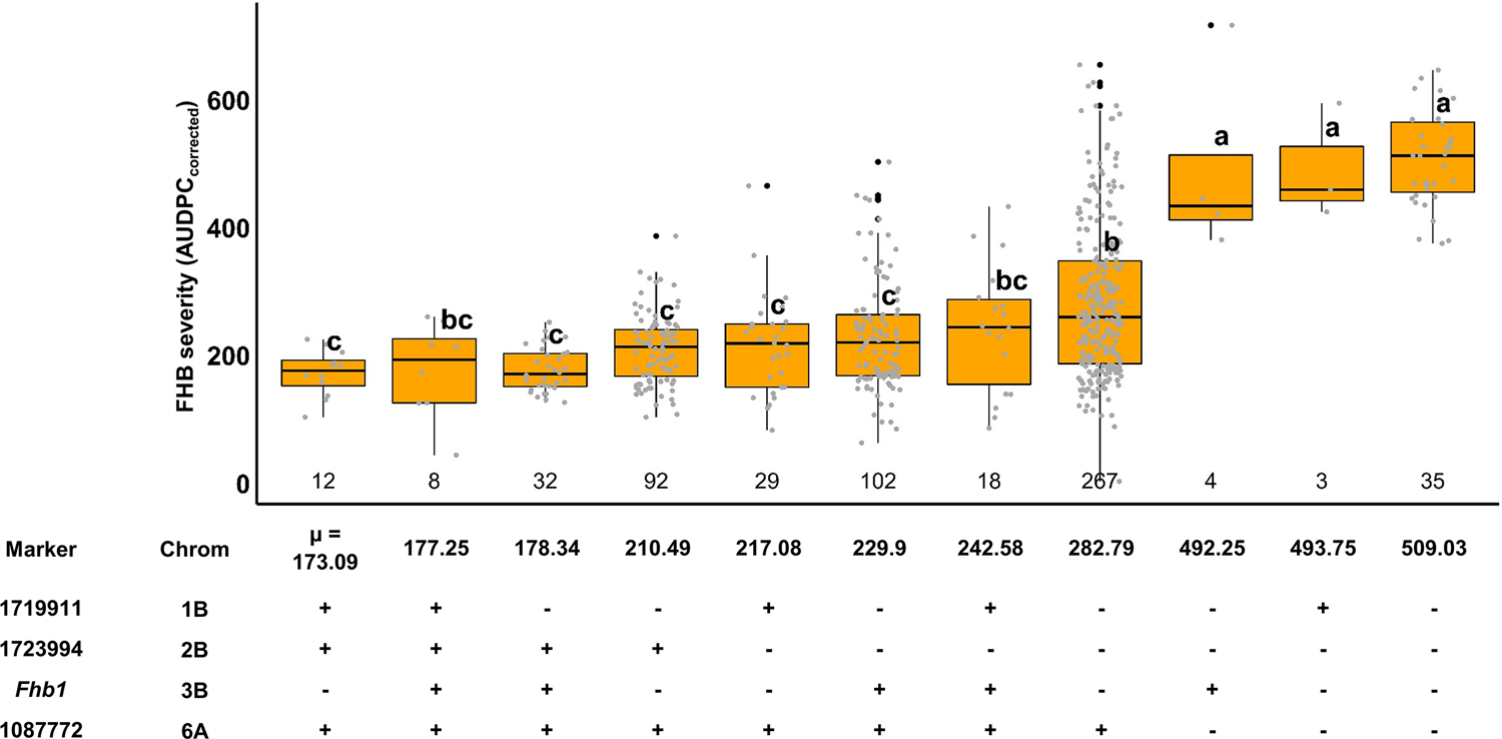
Boxplots of best linear unbiased estimates (BLUEs) for FHB severity (AUDPC_corrected_) for lines carrying different combinations of QTL. Closest marker, chromosome, the number of lines (*n*) for each group shown above the axis line and the mean (*µ*) of FHB severities (AUDPC_corrected_) below the axis line for each group are given. Groups with different index letters are significantly different at *p *< 0.05 based on Tukey test

We investigated the effects of individual QTL and their combinations on FHB severity by grouping the lines according to their genotypes at four associated markers. Lines carrying all three beneficial alleles from the resistance donors, *i.e. Fhb1* as well as the ‘1B and 2B QTL’ derived from *T. diccoccum* and *T. dicoccoides*, had markedly reduced FHB severities (*µ *= 177.25) given that they did not possess the unfavorable allele at the ‘6A QTL’ from durum wheat SZD1029K (Fig. 4). Significant differences were found for groups that carried the adverse ‘6A SZD1029K allele’ irrespective of the allelic status at *Fhb1*, although *Fhb1* carriers showed lower FHB severities (*µ *= 492.25) compared to lines with no resistance QTL (*µ *= 509.03) (Fig. 4).

**Fig. 5:**
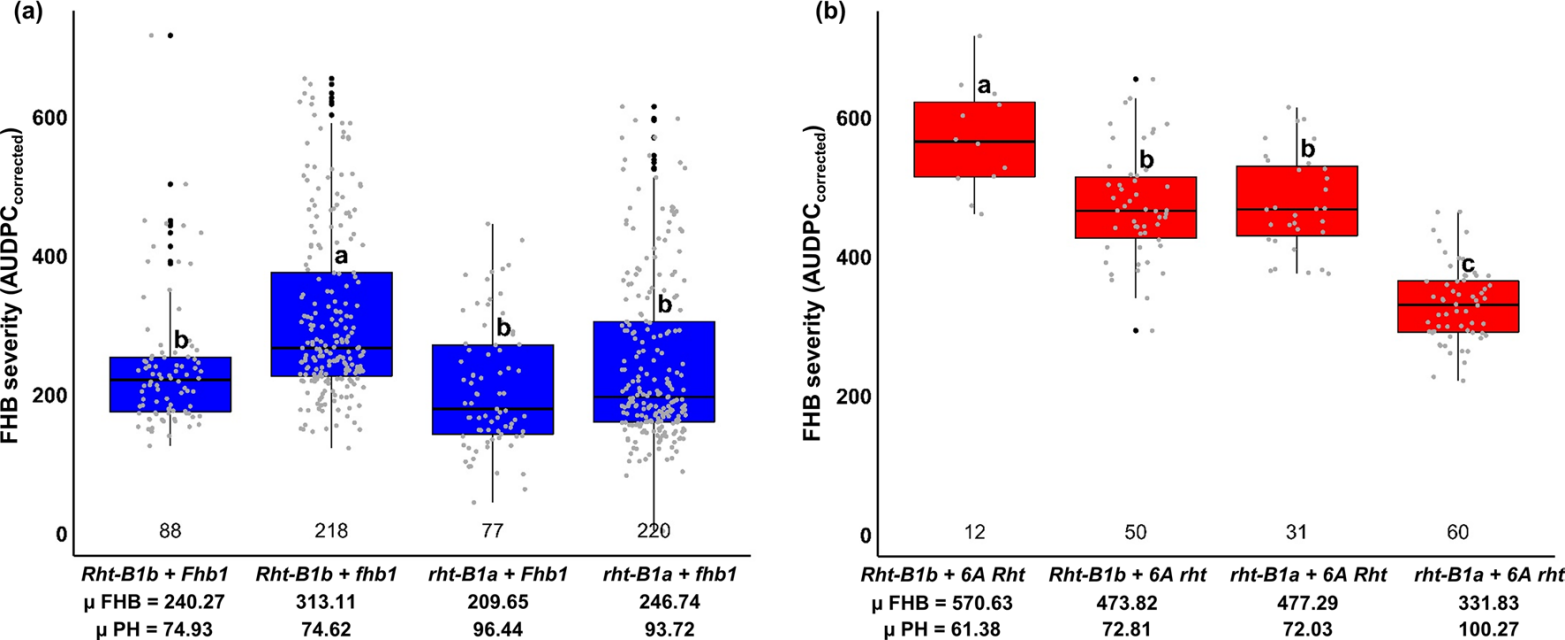
Boxplots of best linear unbiased estimates (BLUEs) for FHB severity (AUDPC_corrected_) for lines carrying different combinations of **a** *Rht-B1* and *Fhb1* from the whole population and **b** *Rht-B1* and the ‘6A QTL’ (6A Rht) for the subpopulations 1, 2 and 3 derived from SZD1029K durum wheat parent. The number of lines for each group is shown above the axis line, the mean (*µ*) of FHB severities (AUDPC_corrected_) and plant height (PH) in cm for each group are given under the axis line. Groups with different index letters are significantly different at *p *< 0.05 based on Tukey test

The effect of *Fhb1* on FHB severity for lines with contrasting *Rht-B1* alleles is shown in Fig. 5a. The favorable allele at *Fhb1* significantly reduced FHB severities for lines carrying the dwarfing allele *Rht-B1b*, whereas its effect was not significant for lines having the tall *rht-B1a* allele although these lines also showed lower disease severities. In subpopulations 1, 2 and 3, two loci, *Rht-B1* and the ‘6A QTL’, equally affected plant height and FHB severity. Lines carrying both semi-dwarfing genes were the shortest and had significantly higher severities, while lines with the ‘tall alleles’ at both loci were the most resistant and tallest (Fig. 5b).

## Discussion

### Anthesis date and plant height modify FHB severities

Although substantial efforts have been made in breeding FHB-resistant durum wheat, the resistance levels among registered cultivars and elite breeding germplasm range from moderately to highly susceptible (Miedaner and Longin 2014; Haile et al. 2019; Steiner et al. 2019; Bentivenga et al. 2020). Due to the lack of resistant elite germplasm, introgression of resistance QTL from wild and cultivated relatives has become an important breeding strategy. In the present study, we investigated a large durum wheat population of 603 lines derived from crosses of such resistance donors with elite durum wheat cultivars. The material was evaluated under high disease pressure in artificially inoculated and humidity-controlled field trials over several years. Despite the lack of highly resistant lines, a wide variation for FHB resistance was observed, including many moderately resistant lines. Anthesis date and plant height strongly influenced FHB severities resulting in pronounced trait correlations. Especially temperature and humidity around anthesis influence FHB infection and disease development, and a warm and humid environment during anthesis promotes infections and accelerates disease spreading (Pugh et al. 1933; Parry et al. 1995; Kriss et al. 2010). Consequently, fluctuations in these parameters during the anthesis period of the investigated material can cause ambiguous associations between FHB resistance and anthesis date and colocalization of QTL which may not be genetically controlled (He et al. 2016; Moreno-Amores et al. 2020; Thambugala et al. 2020; Franco et al. 2021). To correct for these spurious dependencies, Moreno-Amores et al. (2020) established a best-subset multiple linear regression analysis comprising anthesis date plus accumulated thermal time variables, which revealed higher prediction accuracies for FHB severity across years compared to uncorrected data or linear regression on anthesis date only.

We analyzed FHB severity field data collected over nine years with partly high temperature variations within and between years which could be balanced in their effects on FHB infestation by considering cumulative degrees for each evaluation date in addition to the anthesis date. This adjustment reduced the correlation between FHB severity and anthesis date to a negligible level, and resulted in the detection of FHB resistance QTL that showed no significant effect for anthesis date.

The other relationship found in this study between FHB severity and plant height is also very well-known, with taller plants tending to be more resistant to FHB (Mao et al. 2010; Haile et al. 2019; Buerstmayr et al. 2020). Various hypotheses have been formulated to explain this association including microclimatic conditions exposing taller plants to less humid conditions (Mesterhazy 1995), taller plants having a larger distance to the soil surface and the inoculum source from infected debris (Hilton et al. 1999) as well as genetic causes like pleiotropy or linkage of QTL underlying plant height and FHB resistance (Draeger et al. 2007; Srinivasachary et al. 2008; Buerstmayr et al. 2020). Especially the widely distributed dwarfing allele *Rht-B1b* at the reduced height locus *Rht-B1* (syn. *Rht1*) has been repeatedly reported to strongly increase FHB susceptibility in durum wheat (Buerstmayr et al. 2012; Miedaner et al. 2017; Prat et al. 2017; Steiner et al. 2019; Bentivenga et al. 2020; Ruan et al. 2020; Sari et al. 2020). Consistent with these observations, the *Rht-B1* locus also influenced plant height and FHB resistance in our population. The use of plant height as a covariate in the GWAS model adjusted for this known dependency and revealed seven significant associations for FHB severity. This result confirms the quantitative nature of FHB resistance as found in numerous studies (Haile et al. 2019; Venske et al. 2019; Buerstmayr et al. 2020; Ma et al. 2020).

### *Fhb1* and QTL from emmer wheat increase FHB resistance

The most significant association was detected for the *Fhb1-*specific marker KASP46 confirming the successful introgression of the major resistance QTL derived from hexaploid wheat and its effectiveness in diverse durum wheat backgrounds. *Fhb1* had a beneficial effect on improving FHB resistance but did not influence anthesis date nor plant height. This most prominent and best validated FHB resistance QTL has been originally mapped in the Chinese spring wheat line Sumai-3 (Waldron et al. 1999; Anderson et al. 2001) and confers resistance to fungal spread. This resistance component is most reliably evaluated after single spikelet inoculation by assessing the disease spread from the infection point, but also significantly reduces FHB severities under conditions that mimic natural epidemics as revealed by several studies in bread wheat (Miedaner et al. 2006; Von der Ohe et al. 2010; Salameh et al. 2011). Prat et al. (2017) first reported the successful introgression of *Fhb1* into durum wheat by analyzing three bi-parental durum wheat populations after single spikelet inoculation in the greenhouse and spray inoculation in the field. Ruan et al. (2020) confirmed the effectiveness of *Fhb1* in durum wheat after natural infection in an association mapping panel comprising durum wheat lines from the above-mentioned populations. The current study validated these findings in diverse genetic backgrounds regardless of the statistical procedure (correction for anthesis date and/or plant height) suggesting that *Fhb1* significantly and consistently reduces FHB severity in durum wheat even under field conditions. These results underlined thus the usefulness of *Fhb1* in practical FHB resistance breeding programs for durum wheat.

Although a single QTL like *Fhb1* can improve FHB resistance significantly, pyramiding resistance genes is important to increase the level and the durability of resistance (Mundt 2018; Jia et al. 2018; Buerstmayr et al. 2020; Dai et al. 2022). Hence, lines that combined *Fhb1* and resistance QTL derived from *T. dicoccum* line Td161 on chromosome 1B and QTL on chromosome 2B inherited from *T. dicoccoides* accessions Mt. Hermon#22 and Mt. Gerizim#36 showed a profound decrease in FHB severities. These genetic resources have thus the potential to improve FHB resistance in durum wheat, which is in line with previously published studies on *T. dicoccum* line Td161 (Buerstmayr et al. 2012) and *T. dicoccoides* accessions Mt. Hermon#22 and Mt. Gerizim#36 (Gladysz et al. 2007; Buerstmayr et al. 2013). FHB resistance QTL have been mapped to similar regions both in durum wheat and hexaploid wheat on chromosome 1BL (Skinnes et al. 2010; Szabó-Hevér et al. 2014; Ruan et al. 2020) and chromosome 2BS (Giancaspro et al. 2016; Ruan et al. 2020), but low mapping resolution and large linkage blocks comprising thousands of genes do not allow speculations regarding similar genetic control.

Nevertheless, additional populations segregating for these resistance QTL are under development and will be employed to validate these QTL to facilitate their usage in practical durum wheat breeding.

### Unfavorable alleles in elite durum wheat—the '*Rht* problem'

The generally higher susceptibility of durum wheat in contrast to bread wheat has led to speculations about the presence of susceptibility factors and resistance suppressors in durum wheat (Kishii et al. 2005; Fakhfakh et al. 2011; Ghavami et al. 2011; Zhu et al. 2016). In this study, we discovered four loci in elite durum wheat that significantly increased FHB severity. The highly susceptible and very short elite line SZD1029K had the unfavorable alleles for all these loci and was furthermore the exclusive carrier of the susceptibility allele at the locus on chromosome 6A, which also simultaneously shortened plant height and delayed anthesis. This locus was also identified by Prat et al. (2017), who analyzed a DBC480 by SZD1029K mapping population and found a similar effect of the SZ1029K allele on FHB resistance, plant height and anthesis date. The concomitant effect on anthesis date may explain the detection of the QTL in this study despite using plant height as a covariate and suggests that the contribution of the ‘6A QTL’ to susceptibility is partially independent from plant height.

At least four gibberellin-sensitive dwarfing genes have been described for this chromosomal region of 6AL: *Rht14, Rht16, Rht18, and Rht24*; whether these genes are alleles of the same locus or reflect multiple loci has not been fully elucidated (Haque et al. 2011; Würschum et al. 2017; Ford et al. 2018; Vikhe et al. 2019; Duan et al. 2022; Tian et al. 2022). *Rht14, Rht16, Rht18* are used in durum breeding programs and were generated from artificial mutation in durum wheat (Konzak et al. 1984; Scarascia Mugnozza et al. 1993; Ford et al. 2018). Recently, Tian et al. (2022) reported that *Rht24b* originated in wild emmer and encodes a gibberellin 2-oxidase, *TaGA2ox-A9,* conferring higher expression of *TaGA2ox-A9* in stems, leading to a reduction of bioactive gibberellin in stems but an elevation in leaves at the jointing stage.

These gibberellin-sensitive dwarfing genes are genetically and functionally distinct from the widely used gibberellin-insensitive semi-dwarfing gene *Rht-B1b*. Noticeably *Rht-B1b* has been reported to strongly increases FHB susceptibility (Prat et al. 2014; Haile et al. 2019), while *Rht24b* showed no negative effect on FHB resistance suggesting its preferred use in FHB resistance breeding (Würschum et al. 2017; Herter et al. 2018; Miedaner et al. 2022).

However, in our study, the semi-dwarfing genes at *Rht-B1* and the 6A locus showed similar negative effects on FHB resistance. We assume that due to the large variation in plant height, with differences of about 1 m between the shortest and tallest lines, part of the negative effect on FHB severity can be attributed to plant height per se. Using spray inoculation and mist irrigation, the heads of short lines tended to remain wetter and are therefore under more severe infection pressure than the head of taller lines. This issue might have masked the differential effect of *Rht-B1b* and the gibberellin-sensitive *Rht* gene on chromosome 6A on FHB severity in our study.

Nevertheless, durum wheat breeders should be careful when selecting loci that reduce plant height and compensate possible negative effects by introgression of resistance QTL like *Fhb1*.

The major concern associated with FHB is the potential contamination with mycotoxins, specifically deoxynivalenol (DON). Yet, quantifying DON content is more expensive and technically more challenging compared to assessing FHB disease symptoms on the plants. For bread wheat, a comprehensive meta-analysis conducted by Paul et al. (2005) examined the relationship between visual disease evaluations in the field and DON content. Analyzing 163 studies, the results showed overall positive and significant associations, with correlation coefficients ranging from *r *= 0.62 to *r *= 0.53 between FHB field assessments and DON contents. Similar results were observed in durum wheat. Zhao et al. (2018) assessed FHB severity and DON content in a bi-parental mapping population, revealing highly significant associations (*r *= 0.75**) and the identification of the same QTL associated with FHB resistance and DON accumulation. Additionally, Scarpino and Blandino (2021) reported in their durum wheat study that cultivars exhibiting lower susceptibility to FHB also displayed reduced occurrence of DON, as well as its modified forms (DON-3-O-glucoside), zearalenone, and emerging mycotoxins such as moniliformin and enniatins. Therefore, based on these collective findings, it can be inferred that the developed moderately resistant durum wheat lines are likely to exhibit lower levels of DON content as well. Finally, and most important for practical breeding, we have developed moderately resistant and short (plant height of about 65 cm) durum wheat lines that represent breeding-relevant genetic resources and are readily shared within the durum wheat community.

## Conclusion

Harnessing the potential of genetic resources can enlarge the durum wheat gene pool and accelerate the genetic improvement of FHB resistance. We, therefore, conducted genome-wide association mapping to decipher the genetic architecture of FHB resistance in a large durum wheat panel with multiple parents. It was thereby pivotal to adjust the FHB severity scorings for temperature fluctuations during the anthesis period to prevent spurious associations. The plant material itself was derived from crosses between durum wheat elite germplasm and resistance donors carrying introgressions of resistance alleles from *T. aestivum*, *T. dicoccum* and *T. dicoccoides* in durum wheat background. Pyramiding the favorable alleles from these resistance donors at the detected marker-trait associations revealed that such a strategy can achieve an increase in FHB resistance. Moreover, identification of susceptibility alleles in the elite durum wheat germplasm and the selection against these alleles can further boost resistance levels. Hence, combining multiple resistance QTL from wild and cultivated relatives of durum wheat can be considered a promising strategy in breeding, which will lead to the development of new cultivars with increased FHB resistance in the long term.

## Supplementary Information

Below is the link to the electronic supplementary material.

Supplementary file1 (XLSX 26560 KB)

Supplementary file2 (DOCX 2726 KB)

## Data Availability

The plant material and datasets employed in this study are available from the corresponding author on reasonable request.
