# Supplementary material for: Pyramiding Fusarium head blight resistance QTL from T. aestivum, T. dicoccum and T. dicoccoides in durum wheat
Source: Theor Appl Genet. 2023 Aug 28;136(9):201. doi: 10.1007/s00122-023-04426-7 (PMC10462738; doi:10.1007/s00122-023-04426-7)
Supplement: Supplementary file 2 — Supplementary file2 (DOCX 2726 KB) [file 122_2023_4426_MOESM2_ESM.docx]

**Online Resource 2**

**Article title:** Pyramiding Fusarium head blight resistance QTL from *T. aestivum*, *T. dicoccum* and *T. dicoccoides* in durum wheat

**Journal:** Theoretical and Applied Genetics

**Authors:** Rizky Pasthika Kirana, Sebastian Michel, Jose Moreno-Amores, Noemie Prat, Marc Lemmens, Maria Buerstmayr, Hermann Buerstmayr, Barbara Steiner

**Name, affiliation, and email of corresponding author:**

Barbara Steiner, University of Natural Resources and Life Sciences, Vienna, Department of Agrobiotechnology (IFA-Tulln), Institute of Biotechnology in Plant Production, Konrad-Lorenz-Straße 20, 3430 Tulln, Austria; [barbara.steiner@boku.ac.at](mailto:barbara.steiner@boku.ac.at)

**Figure S1** Scatter plots and marginal histograms of frequency distribution of best linear unbiased estimates (BLUEs) across years for FHB severity against anthesis date and plant height

**Figure S2** Scatter plot of genome-wide linkage disequilibrium decay

**Figure S3** Scatter plots of linkage disequilibrium decay for individual chromosomes

**Figure S4** Manhattan and Q-Q plots of SNPs associations with FHB resistance using best linear unbiased estimates (BLUEs) across years of FHB severity (AUDPC_uncorrected_) (a) without plant height as a covariate and (b) with plant height as a covariate in the GWAS model.


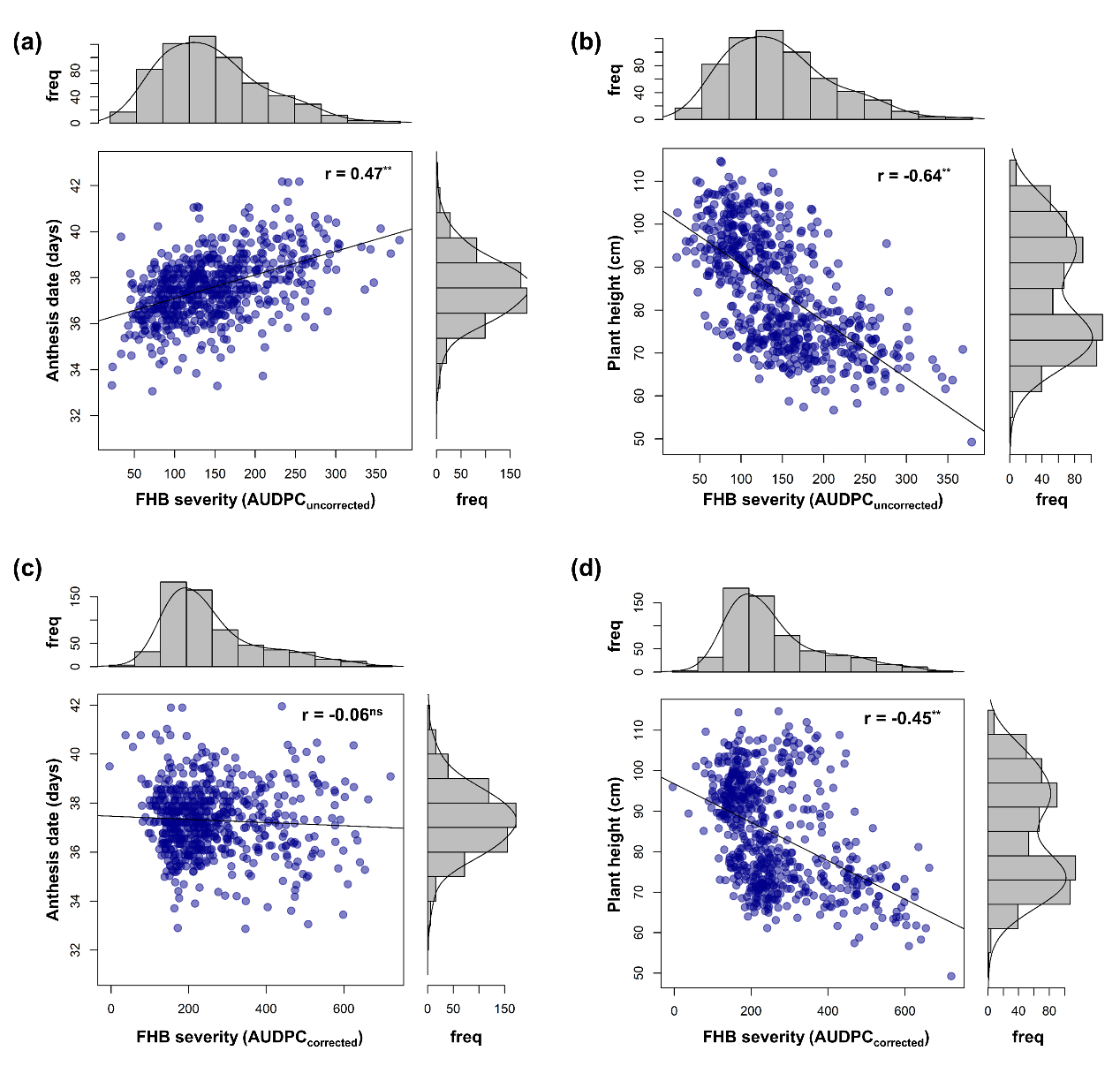


**Supplementary Fig. S1** Scatter plots and marginal histograms of frequency distribution of best linear unbiased estimates (BLUEs) across years for FHB severity (AUDPC_uncorrected_) against (a) anthesis date (number of days from May 1^st^), (b) plant height (cm) and FHB severity (AUDPC_corrected_) against (c) anthesis date (number of days from May 1^st^); (d) plant height (cm)


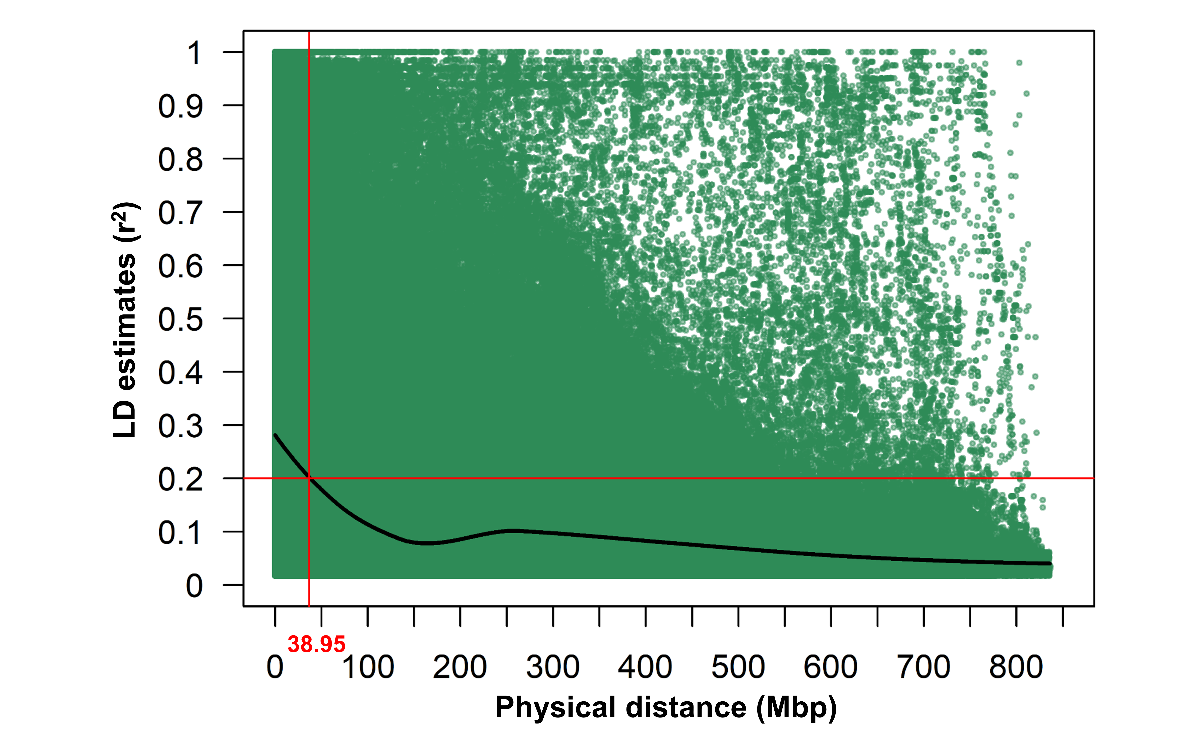


**Supplementary Fig. S2** Scatter plot of genome-wide linkage disequilibrium (LD) (r^2^) versus physical distance (Mbp) for the 603 durum wheat lines based on 13,640 markers. The intersection of the fitted locally weighted polynomial regression-based (LOESS) curve with the critical LD value r² = 0.2 is indicated.


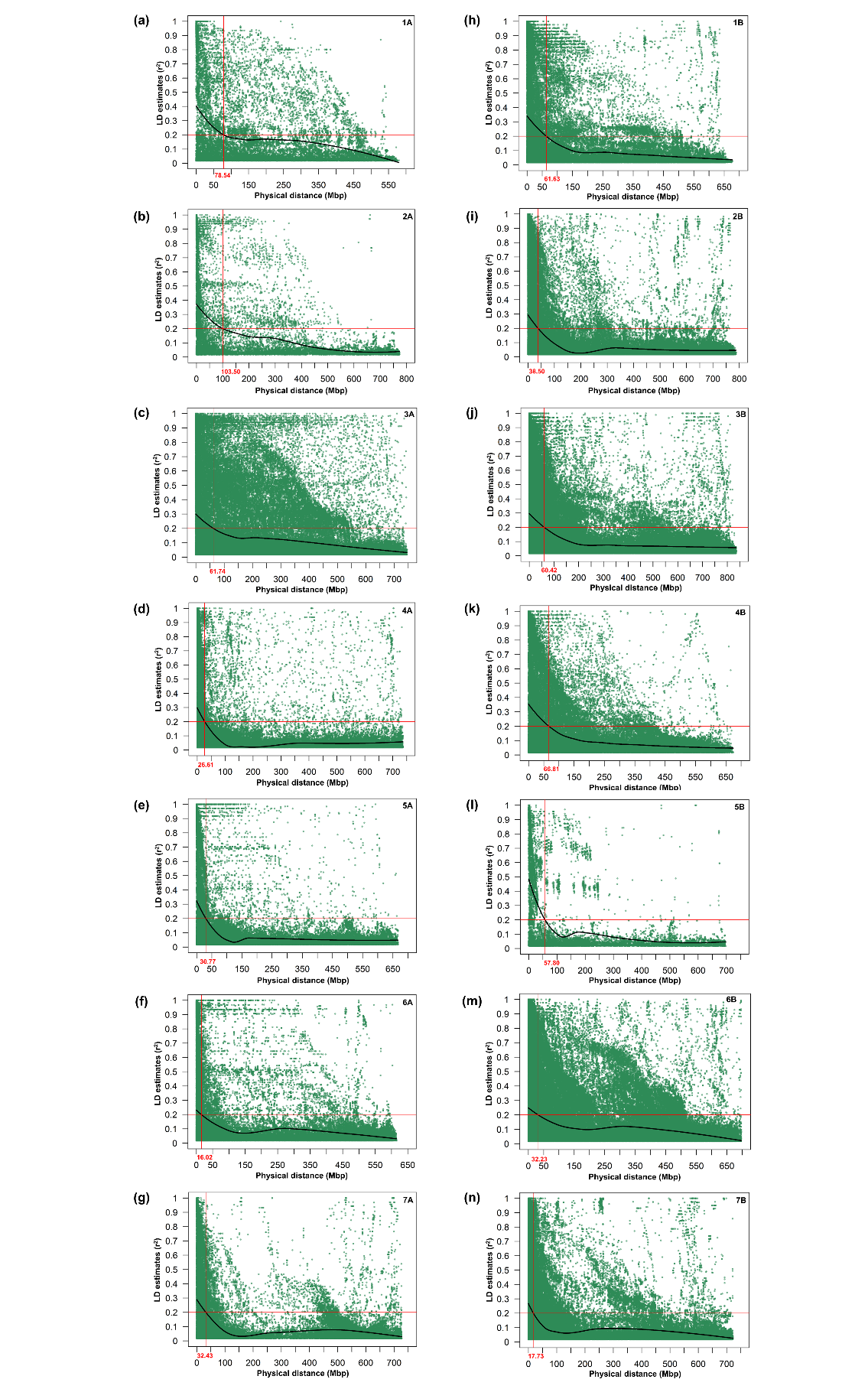


**Supplementary Fig. S3** Scatter plots of linkage disequilibrium (LD) (r2) of individual chromosomes (a to n) versus physical distance (Mbp) for the 603 durum wheat lines based on 13,640 markers. The intersections of the fitted locally weighted polynomial regression-based (LOESS) curve with the critical LD value r² = 0.2 are indicated.


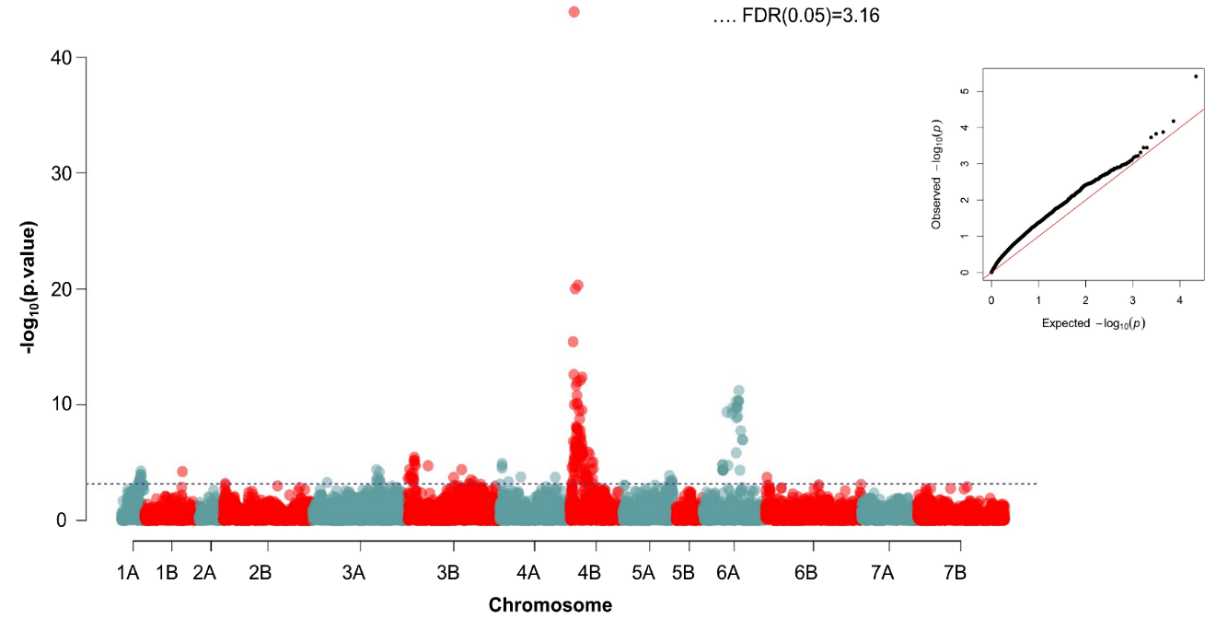


**Supplementary Fig. S1b**Markers density and distribution on durum wheat **chromosome** of durum wheat genotypes

**Supplementary Fig. S1a**Markers density and distribution on durum wheat **subgenome** of durum wheat genotypes

**(b)**

**(a)**


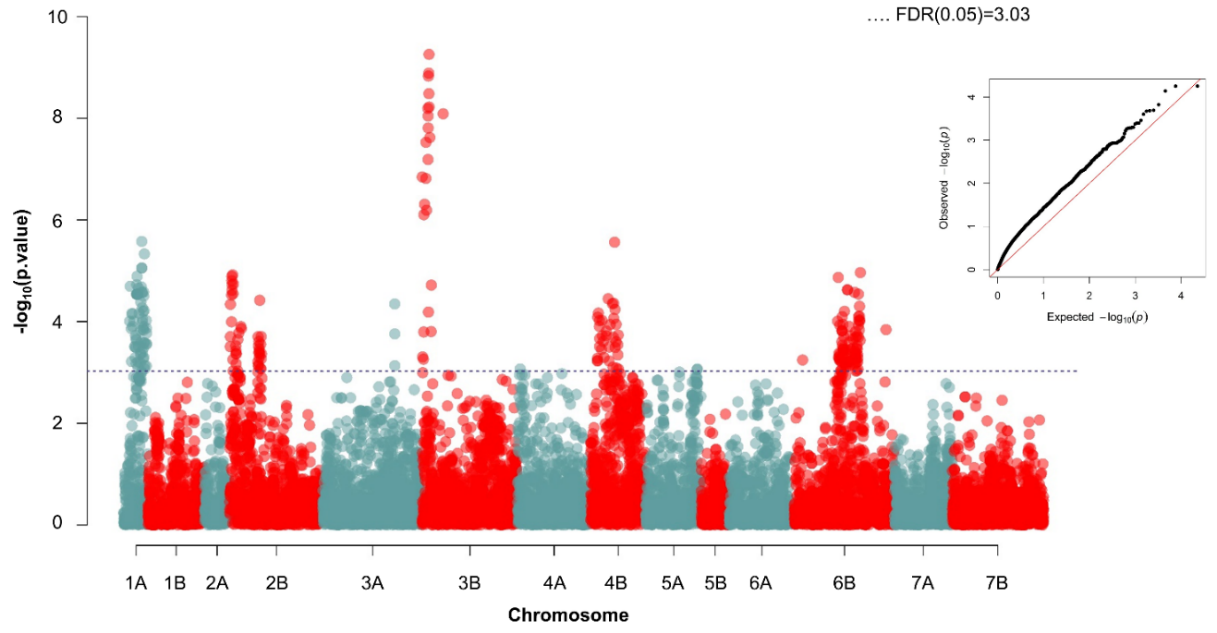


**Supplementary Fig. S4** Manhattan and Q-Q plots of SNPs associations with FHB resistance using best linear unbiased estimates (BLUEs) across years of FHB severity (AUDPC_uncorrected_) (a) without plant height as a covariate and (b) with plant height as a covariate in the GWAS model. The horizontal dotted line shows the −log10(p) value with FDR 5% significant threshold with FHB severity
